# The importance of definitions in crystallography

**DOI:** 10.1107/S2052252524004056

**Published:** 2024-05-28

**Authors:** Olga Anosova, Vitaliy Kurlin, Marjorie Senechal

**Affiliations:** a Computer Science Department and Materials Innovation Factory University of Liverpool Liverpool United Kingdom; b Clark Science Center Smith College USA; IRCP Chimie-ParisTech, France

**Keywords:** phase problem, structure prediction, materials modeling, crystal definition

## Abstract

This paper was motivated by the articles ‘Same or different – that is the question’ in *CrystEngComm* (July 2020) and ‘Change to the definition of a crystal’ in the *IUCr Newsletter* (June 2021). Experimental approaches to crystal comparisons require rigorously defined classifications in crystallography and beyond. Since crystal structures are determined in a rigid form, their strongest equivalence in practice is rigid motion, which is a composition of translations and rotations in 3D space. Conventional representations based on reduced cells and standardizations theoretically distinguish all periodic crystals. However, all cell-based representations are inherently discontinuous under almost any atomic displacement that can arbitrarily scale up a reduced cell. Hence, comparison of millions of known structures in materials databases requires continuous distance metrics.

## Abstract 2

This paper proposes a rigorous definition of a periodic structure as an equivalence class of periodic sets under rigid motion, which is a composition of translations and rotations.

## 1. Motivations for new definitions in crystallography

Mathematical crystallography – including the classification of lattices, unit cells, crystal classes *etc.* – by symmetries has a long and rich history. But classical mathematical crystallography, grounded largely in group theory, came before the computer age and needs updating in our era of massive data.

This paper does not reinvent the wheel but extends the discrete concepts to a new continuous domain in the language of present-day crystallography for present-day crystallographers.

The entry ‘A crystal’ appeared in the *IUCr Online Dictionary of Crystallography* (IUCr, 2021 ▸) in 1992 and has since been modified slightly. We propose updates to fill past gaps and meet present needs. The latest direct-space definition (Chapuis, 2024*a* ▸) by the Commission on Crystallographic Nomenclature (CCN) says that ‘a solid is a crystal if its atoms, ions and/or molecules form, on average, a long-range ordered arrangement. In most crystals, the arrangement is a periodic array that is governed by the rules of translational symmetry.’

In this paper, a *crystal* is a periodic crystal, so we postpone similar developments for non-periodic materials including quasicrystals and amorphous solids to future work (see Senechal, 1996 ▸). The definition quoted above (Brock, 2021 ▸) for a periodic crystal means that the set of all atoms is preserved under all lattice translations. Since periodic crystals, lattices and unit cells are often confused (Nespolo, 2015 ▸) or used interchangeably, we provide rigorous definitions in Section 2.

The next step is to clarify which periodic crystals should be considered the *same* in order to reliably compare crystals. Below, we quote the paper ‘Same or different – that is the question’ (Sacchi *et al.*, 2020 ▸). It is correct scientific practice ‘to report measurable quantities with an error’ because all real measurements are noisy. However, if one claims that ‘… two dimensions are considered the same if their values fall within the accepted error or standard deviation’, quoted from section 2.1 in Sacchi *et al.* (2020 ▸), then an axiomatic approach logically implies that all dimensions (meaning measurements of unit-cell parameters in this case) should be the ‘same’. This continuum paradox (Hyde, 2011 ▸) states that many small changes (indistinguishable from 0) can lead to a big overall change.

For any fixed small error 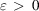, if we call any real number 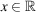 indistinguishable from (considered the same as) all numbers within an interval 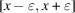, then 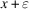 is the same as all numbers from 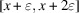, which makes *x* the same as any number within 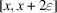, similarly within 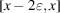 if we replace ɛ with 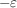. Continuing this logical argument further, any number *y* becomes indistinguishable from *x* in 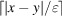 steps, which is the smallest integer larger than or equal to 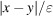. This argument is formalized in terms of an equivalence below.

Definition 1 (equivalence relation) A binary relation *A* ∼ *B* between objects of any kind is called an *equivalence* (Raczkowski & Sadowski, 1990 ▸) if these axioms hold:

(1) *reflexivity*: *A* ∼ *A*, so any object *A* is equivalent to itself;

(2) *symmetry*: for any objects *A*, *B*, if *A* ∼ *B* then *B* ∼ *A*;

(3) *transitivity*: for any *A*, *B*, *C*, if *A* ∼ *B*, *B* ∼ *C* then *A* ∼ *C*.

Definition 1[Statement definition1] is important because any well defined classification into disjoint classes requires an equivalence relation. Indeed, the *equivalence class* of any object 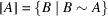 is the set of all objects *B* equivalent to *A*. The transitivity axiom implies that if the classes of *A*, *C* share a common object *B*, these classes coincide, *i.e.* [*A*] = [*C*]. Hence Definition 1[Statement definition1] guarantees that all equivalence classes are disjoint. For any fixed 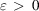, the binary relation *x* ∼ *y* defined by 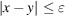 on real numbers fails the transitivity axiom because 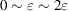 but 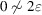.

If we enforce the transitivity so that *x* ∼ *z* if there is *y* such that *x* ∼ *y* ∼ *z*, this transitive extension makes all real numbers equivalent by putting them into a single equivalence class. Equality is an example of equivalence because any number can be written in many different forms: 0.5 = 1/2 = 50% = 1:2.

If the axioms of Definition 1[Statement definition1] such as the transitivity are not satisfied, the resulting classes can overlap and become dependent on manually chosen parameters, see Zwart *et al.* (2008 ▸). All relations between lattices and crystals that led to 7 crystal systems, 14 Bravais classes and 230 space-group types are equivalences satisfying the axioms. A space-group type is a class of space groups under *isomorphism*, which is a bijection respecting the group operation, see Nespolo *et al.* (2018 ▸).

The most important practical motivation to agree on the main equivalences between crystals is the ongoing crisis of fake data in crystallography (Gavezzotti, 2022 ▸), which has caught attention of journalists (Chawla, 2024 ▸). Indeed, scientists could stop the ‘paper mills’ (Bimler, 2022 ▸) that publish hundreds of articles and thousands of crystal structures, many of which are under investigation for data integrity (Francis, 2023 ▸).

In November 2023, two *Nature* papers described the recent ‘big data’ attempts at generating crystal structures. The first paper (Merchant *et al.*, 2023 ▸) reported the GNoME database of 384+ thousand ‘stable’ predicted structures. The chemists found ‘scant evidence for compounds that fulfill the trifecta of novelty, credibility and utility’ (Cheetham & Seshadri, 2024 ▸).

The autonomous A-lab (Szymanski *et al.*, 2023 ▸) claimed to have synthesized 43 new materials from GNoME. The review by Leeman *et al.* (2024 ▸) concluded that ‘none of the materials produced by A-lab were new: the large majority were misclassified, and a smaller number were correctly identified but already known’. Section 6 will complement these conclusions by identifying thousands of duplicates in GNoME.

## 2. Common confusions with cells, lattices and crystals

In our papers (Widdowson *et al.*, 2022 ▸; Widdowson & Kurlin, 2022 ▸), we introduced a unit cell, lattice and periodic crystal in a single definition without explaining their logical dependencies. This approach suffices for expert mathematicians, but since many publications confuse lattices not only with crystals but also with cells, we clarify the differences here.

We are grateful to Massimo Nespolo for highlighting the differences between a periodic lattice and a crystal structure (Nespolo, 2019 ▸). Confusing these concepts led to the terms ‘lattice energy’ and ‘lattice defects’, which should be better called ‘structural energy’ and ‘structural defects’. Since section 2 in Nespolo (2019 ▸) defined ‘the lattice of a crystal structure… as a collection of vectors expressed as a linear combination of *n* linearly independent vectors’, we start from the more basic concepts of a basis and a lattice without requiring a crystal structure whose definition needs the pre-requisite concept of a lattice.

Definition 2 (basis and ordered basis) (*a*) A *basis* of 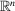 is an unordered set of *n* vectors 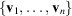 in 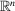 that are ‘linearly independent’, *i.e.*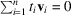 if, and only if 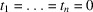.

(*b*) An *ordered basis* of 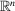 is a basis whose vectors 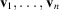 are ordered. Equivalently, any vector 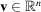 can be expressed as a linear combination 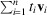 for unique 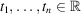.

For example, the vectors **v**_1_ = (1, 0), **v**_2_ = (0, 1) form a basis of 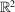 because any vector 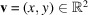 is uniquely written as the linear combination 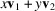. We can write coordinates of any vector 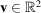 in a unique order only if **v**_1_, **v**_2_ are ordered.

Following our standards of introducing all concepts with an equivalence, these definitions imply that two bases are equivalent if they are equal as sets, while two ordered bases are equivalent if they contain the same vectors in the same order. A basis is often confused with the unit cell defined by this basis.

Definition 3 (the unit cell and lattice defined by a basis) Any unordered basis 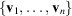 of 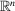 defines a *unit cell*: the parallelepiped 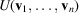 consisting of all linear combinations 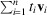 with real coefficients 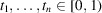. This basis also generates the *lattice*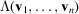 consisting of all linear combinations 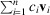 with integer coefficients 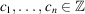.

**Figure 1:**
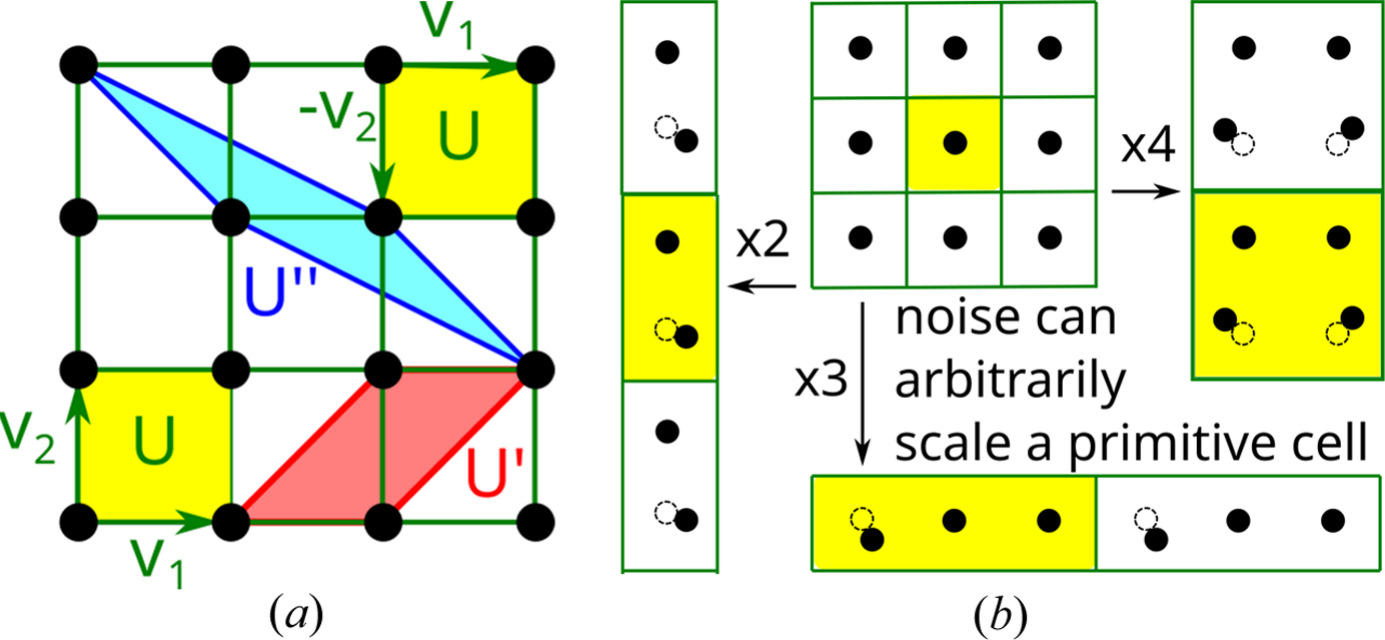
Left: an infinite number of cells generates the same square lattice. Right: almost any perturbation breaks the symmetry and discontinuously scales a primitive cell.

Thus a unit cell is a ‘box’, whereas a lattice is a discrete point set. Fig. 1 ▸ (left) shows that the square cells defined by the orthonormal bases 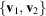 and 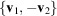 are both unit squares, which differ only by the choice of origin and orientation. The square lattice has infinitely many bases 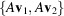, where *A* is a 2 × 2 matrix with integer coefficients and determinant ±1.

For the unit cell 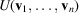, we excluded the values *t_i_* = 1 so that all translations of 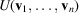 by vectors 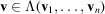 tile 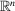 without overlaps. The notation 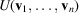 highlights that a unit cell is defined by a basis alone. For now, we consider unit cells (and lattices) equivalent (in the strictest possible sense) if they are equal as sets of points. The map {bases}
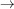
{unit cells} is not invertible because a corner of a unit cell should be chosen for an origin. Fixing one of 2^*n*^ corners is equivalent to choosing *n* signs of ordered basis vectors 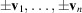. So we cannot uniquely identify an ordered basis from a unit cell without making one of 2^*n*^ choices.

Definition 3[Statement definition3] introduced a unit cell and a lattice using only an unordered basis 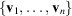 of vectors. Without these vectors, we cannot define their linear combinations.

However, as soon as we need to unambiguously express a point (from a motif below) using fractional coordinates in a basis, this basis 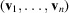 should become ordered so that coordinates of any point are ordered according to the basis.

Definition 4 (motif, periodic point set, periodic crystal) For any ordered basis 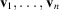 of 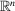, let 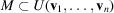 be a finite set *M* of points. We call *M* a *motif*. A *periodic point set*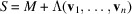 is the set of points 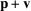 for all 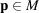 and 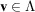. In 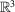, if each point of *M* is an atom or ion with a chemical element and charge, *S* can be called a *periodic crystal*.

In Definition 3[Statement definition3], any periodic crystal has a purely geometric part, which is a periodic set of zero-sized points at all atomic centers, and the physical part of atomic attributes of these points, see the history in Palgrave & Tobin (2021 ▸). Any lattice Λ can be considered a periodic point set whose motif *M* consists of a single point **p**, for example, at the origin of 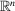. More general periodic crystals, even graphite, have motifs with at least two points and thus are not lattices according to Definition 3[Statement definition3].

Any unit cell can be scaled by a positive integer factor along each basis vector to an extended cell. This additional ambiguity is theoretically resolved by taking a *primitive* cell that is a unit cell of a minimal volume. However, Fig. 1 ▸ (right) shows that any extended cell can be made primitive by a tiny perturbation of a single atom in the initial cell. This discontinuity was reported in 1965, see page 80 in Lawton & Jacobson (1965 ▸), and emerges even in one dimension. For the integer sequence 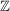, if we shift *m* of every *m* + 1 points by a small 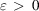, we obtain the periodic sequence 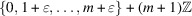 whose every point is ɛ-close to a point of 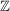 (and vice versa), but the period *m* + 1 can be arbitrarily large after perturbation.

**Figure 2:**
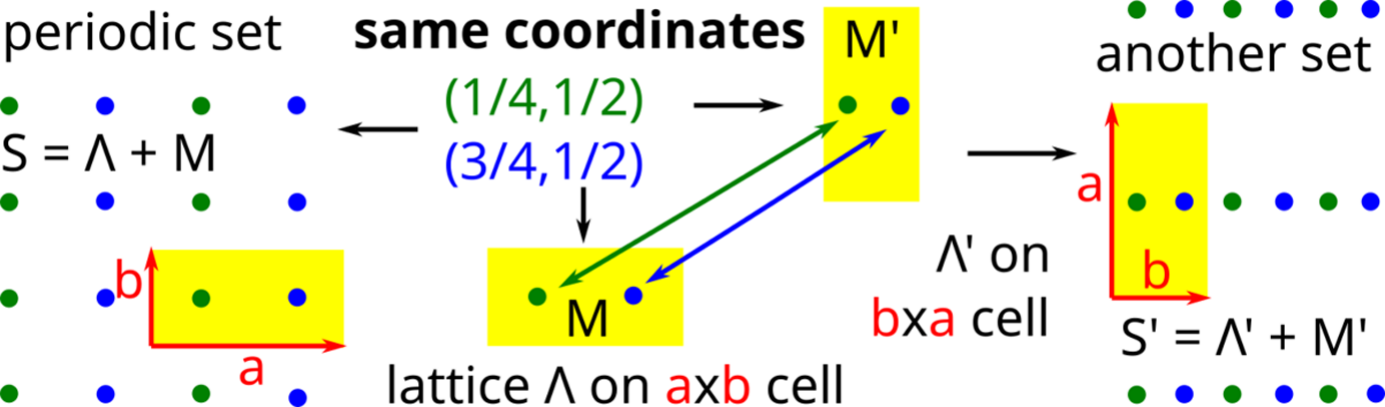
For any *a* > *b* > 0, let the lattices 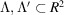 have the unit cells 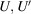 of the rectangular forms *a* × *b*, *b* × *a*, respectively. Any collection of 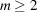 points with fractional coordinates 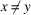 in [0, 1] defines different motifs 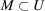 and 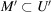. Then the periodic point sets 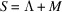, 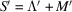 can be arbitrarily different, though their CIFs differ only by swapping the lengths *a*, *b* of the basis vectors.

A crystallographic information file (CIF) contains an ordered basis of vectors in 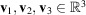 and coordinates of each point 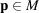 on this basis with the atomic type **p**. The ordered vectors 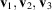 can be uniquely determined from their lengths 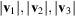 and angles 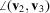, 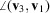, 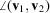. The angles should be ordered according to their opposite vectors. A unit cell without ordered sides (ordered basis vectors) can give rise to different periodic point sets as in Fig. 2 ▸.

**Figure 3:**
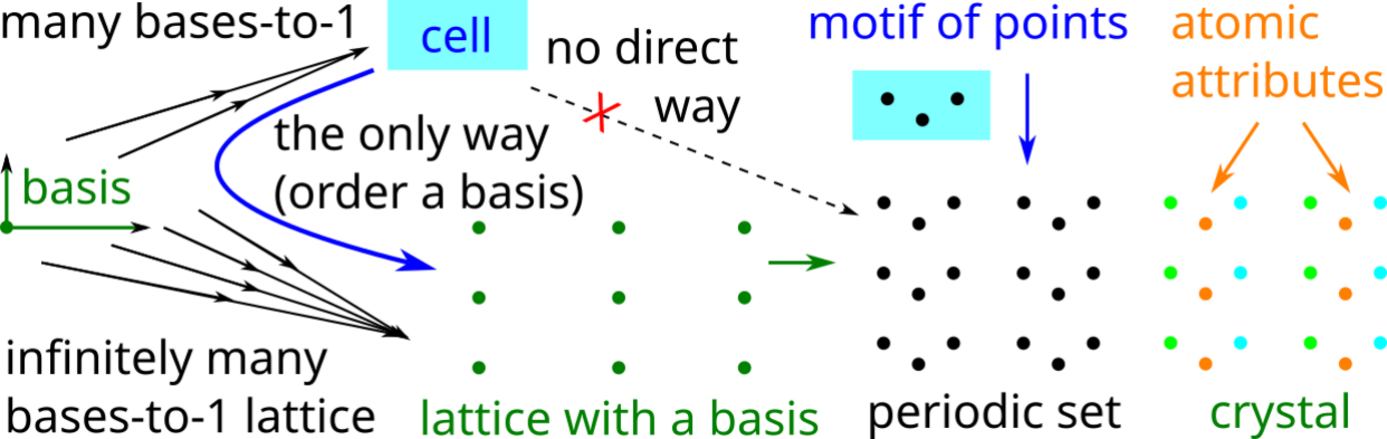
Due to the ambiguity of Fig. 2 ▸, a unit cell *U* with a motif 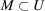 can define a periodic point set only after choosing an ordered basis for *U*. A *periodic point set* is a union of lattices 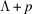 shifted by all 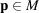. A *periodic crystal* is a periodic set of atoms (points with chemical elements or other attributes).

Ordering basis vectors by their lengths creates another discontinuity if the vectors have equal lengths because small perturbations can change their order. Fig. 3 ▸ summarizes why an ordered basis of 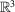 is more convenient for defining a periodic crystal than a unit cell. When the basis 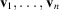 is fixed, we use the shorter notations 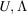 without repeating this fixed basis.

## 3. Rigorous definitions of periodic and crystal structures

In the past, many different equivalence relations between latices and crystals were studied. One of the simplest is by chemical composition or by equality of another property such as density. However, crystals with the same composition (say, diamond and graphite of pure carbon) or with the same density can have many different properties, so these equivalences may not suffice.

Hence we are looking for a stronger equivalence that would guarantee the same physical and chemical properties according to the *structure–property hypothesis* which states that the structure of a material structure should determine all of its properties (Newnham, 2012 ▸).

The IUCr online dictionary (Chapuis, 2024*c* ▸) contains the following entry: ‘crystals are said to be *isostructural* if they have the same structure but not necessarily the same cell dimensions nor the same chemical composition, and with a ‘comparable’ variability in the atomic coordinates to that of the cell dimensions and chemical composition. For instance, calcite CaCO_3_, sodium nitrate NaNO_3_ and iron borate FeBO_3_ are isostructural’. This phrase contains a cycle of ‘structural’ concepts (‘crystals are *isostructural* if they have the same structure’), which should be resolved by defining a *structure*.

If the keyword ‘necessarily’ was omitted above, the reflexivity axiom would fail. Any attempt to define a ‘comparable’ variability with a threshold 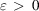 for deviations of cell sizes fails the transitivity axiom. Indeed, by sufficiently applying many tiny deformations, we can convert any given unit cell (with an empty motif, as in Definition 3[Statement definition3]) into any other cell, so the classification under this ‘deviation’ equivalence becomes trivial.

The IUCr online dictionary defines the longer term *crystal structure* as ‘a crystal pattern consisting of atoms’. Both Chapuis (2024*b* ▸) and section 8.1.4 in Hahn (2005 ▸) defined a *crystal pattern* in different words but essentially as a periodic point set in Definition 4[Statement definition4], not considered under rigid equivalence. The word *pattern* as in the area of pattern recognition often refers not to a single object but to a class of objects under an equivalence as we propose in the new Definition 6[Statement definition6] below.

Why do we need an equivalence that distinguishes between all chemical compositions and also close *polymorphs* that have the same composition but different properties? Such an equivalence is important because in the past many HIV patients suffered by unknowingly taking a more stable but less soluble polymorph of ritonavir that was accidentally manufactured instead (Morissette *et al.*, 2003 ▸).

On the other hand, the pointwise coincidence of cells, lattices and periodic sets from Section 2 is too strict. Indeed, shifting the whole motif *M* by a small vector within a fixed unit cell changes all fractional coordinates of atoms in a CIF, but not the actual solid material. We consider all equivalences and comparisons only for ideal periodic crystals and under the same ambient conditions such as room temperature and pressure.

Since crystal structures are determined in a *rigid form*, their strongest and practically important equivalence is rigid motion.

Definition 5 (rigid motion, isometry) A *rigid motion* of 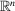 is a composition of translations and rotations. An *isometry* of 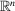 is any transformation that preserves all inter-point distances.

For an ordered basis 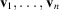 of 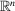, an *orientation* can be defined as the sign of the *n* × *n* determinant with the columns 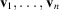. Any orientation-preserving isometry of 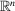 is a rigid motion. Any orientation-reversing isometry of 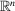 is a composition of one (any) mirror reflection and a rigid motion.

Hence, isometry is a slightly weaker equivalence than rigid motion because mirror images are equivalent under isometry but not always under rigid motion. Since mirror images can be distinguished by a (suitably chosen) sign of orientation, it almost suffices to distinguish crystals only under isometry.

Definition 5[Statement definition5] distinguishes between *isometry*, which makes sense for any metric space with no Euclidean structure, and more restrictive *rigid motion* (orientation-preserving isometry).

The word *motion* is justified by the fact that any rigid motion *f*, which excludes mirror reflections by definition, can be realized through a continuous (motion) family of isometries 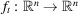, where 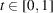, *f*_1_ = *f* and 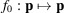 is the identity map. Isometry was called a symmetry operation in section 8.1.3 of Hahn (2005 ▸). Since *symmetry* has a wider meaning in science, we use the more specific concepts of *rigid motion* and *isometry*. The comprehensive books by Engel *et al.* (2004 ▸) and Zhilinskii (2016 ▸) studied lattices through group actions. In this language, any periodic structure from Definition 6[Statement definition6] is a class in the quotient of all periodic point sets under the action of the special Euclidean group 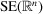 of all rigid motions in 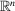.

Definition 6 (periodic and crystal structures) A *periodic structure* is an equivalence class of periodic point sets 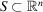 under rigid motion. A *crystal structure* is an equivalence class of periodic crystals with atomic attributes under rigid motion in 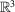.

Section 2 in Nespolo *et al.* (2018 ▸) defined a crystal structure as ‘an idealized periodic pattern of atoms in 3D space using the corresponding coordinates with respect to the chosen coordinate system’. This pattern coincides with a crystal pattern (Chapuis, 2024*b* ▸) in section 8.1.4 of Hahn (2005 ▸) and is a single representative of a periodic structure, introduced as a class of all rigidly equivalent crystals in Definition 6[Statement definition6].

Any explicit use of coordinates for a crystal representation, like in a CIF, requires choosing an ordered basis and a motif of points with fractional coordinates in this basis. Definition 4[Statement definition4] called such objects periodic point sets and periodic crystals. Shifting a motif by a fixed vector changes the description in a CIF but not the real structure which is considered to be a class of equivalent representations

Then a periodic crystal in the sense of the classical cell-based Definition 4[Statement definition4] becomes one of infinitely many coordinate-based representations of a crystal structure in the sense of the new Definition 6[Statement definition6]. Hence crystals are defined as the *same* if all their atoms can be matched by rigid motion. If there is no ideal match, any slightly different structures can be called close rather than ‘the same’ because any tolerance makes the classification trivial.

**Figure 4:**
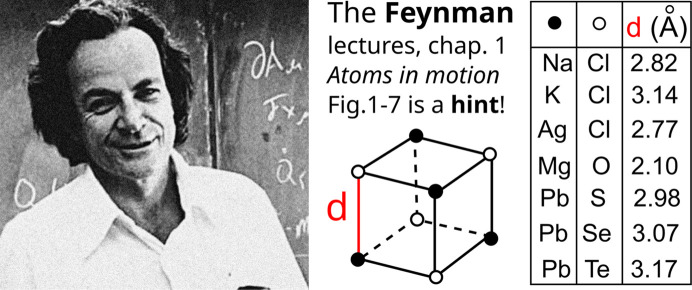
Feynman’s first lecture in Feynman *et al.* (1971 ▸) has a table (redrawn here in a simpler form) of seven cubic crystals that all differ by their periodic structures (purely geometrically) as in Definition 3 after we ignore all chemical elements.

Ignoring atomic attributes maps any periodic crystal to a periodic set of points (atomic centers). Though this projection might seem to lose all chemistry, Richard Feynman gave us a visual hint in his first lecture on atomic theory (Fig. 4 ▸) to compare crystals only by atomic centers without chemical elements.

Despite the apparent simplicity, Definition 6[Statement definition6] brings up a hard problem of efficiently distinguishing periodic structures, which will be stated in Section 6 when defining a few more concepts. A recent and almost complete solution to this problem has made Definition 6[Statement definition6] practically important, especially for detecting thousands of previously unknown near-duplicates in major databases. Sections 4 and 5 will discuss how to distinguish crystal structures and continuously quantify their differences.

## 4. Descriptors versus invariants under a given equivalence

Distinguishing objects under any equivalence relation from Definition 1[Statement definition1] necessarily requires the concept of an invariant. Such a numerical property is often called a feature or descriptor without specifying an equivalence. In the sequel, for simplicity, we use isometry as our main equivalence, denoted by *S* ≃ *Q*. Extensions to rigid motion will need a sign of orientation.

Definition 7 (invariant, complete invariant) A function *I* on periodic point sets is called an *isometry invariant* if any isometric sets *S* ≃ *Q* have *I*(*S*) = *I*(*Q*) or, equivalently if 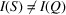 then 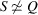. An invariant *I* is called *complete* (injective or separating) if the converse also holds: if 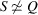 then 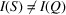.

**Figure 5:**
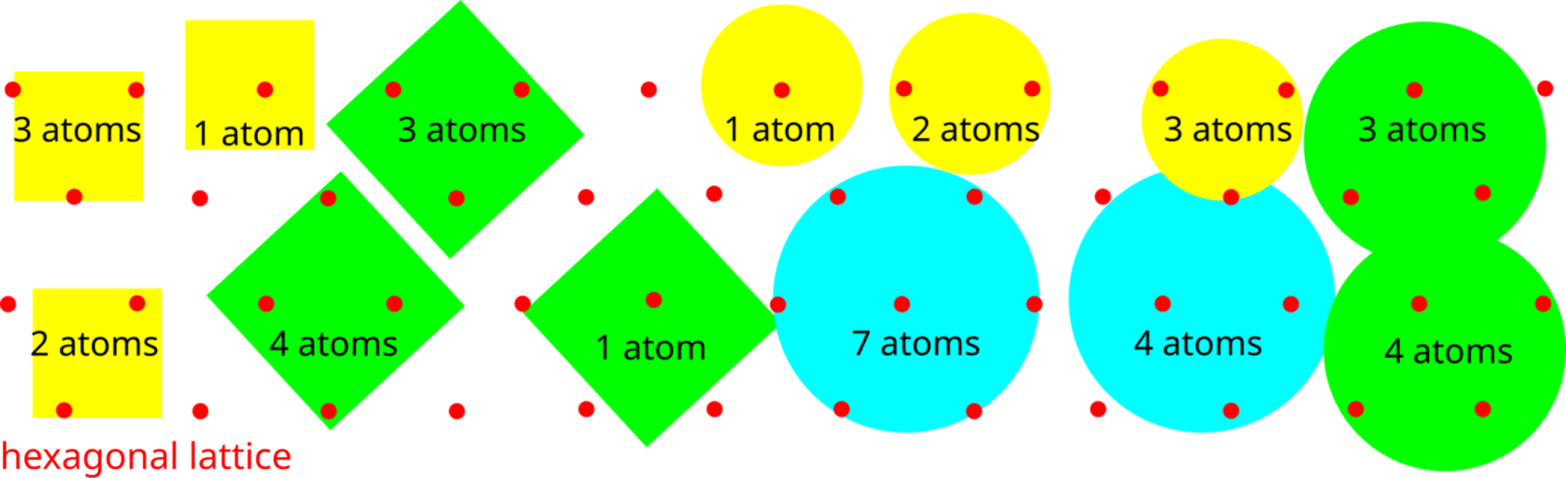
Any periodic set has many non-isometric subsets within boxes or balls of the same cut-off radius. If an original basis is forgotten, it can be hard to reconstruct the initial periodic structure from its arbitrary finite subset.

Though it is very tempting to reduce a periodic point set to a finite subset such as an extended motif, this reduction can lead only to many non-isometric subsets as in Fig. 5 ▸. Hence, there is no simple way to reduce a periodic point set to a single finite subset. Taking finite clouds around every atom in a motif can lead to a complete invariant of periodic point sets under isometry (Anosova & Kurlin, 2021 ▸), but the continuity under perturbations needs careful justifications (Anosova & Kurlin, 2022 ▸).

A simple isometry invariant of a periodic point set *S* is the number *m* of points within a primitive unit cell *U* of *S*. This invariant is weak and cannot distinguish any lattices. A complete (injective or separating) invariant *I* is the strongest possible in the sense that *I* distinguishes all non-isometric sets.

The side-side-side (SSS) theorem from school geometry can be rephrased in terms of invariants by stating that a complete invariant of three unordered points under isometry of 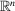 consists of three inter-point distances up to permutations. If all *m* given points are ordered, the *m* × *m* matrix of their pairwise distances is complete under isometry by Schoenberg (1935 ▸).

The case of unordered points is more practical for molecules whose many atoms can be indistinguishable as in the benzene ring. The naive extension of distance matrices to *m* unordered points requires *m*! permutations, which is impractical even for small *m*. Hence the important requirement for invariants is their computability, *e.g.* in polynomial time of the input size.

The invariance condition is the minimal requirement for a descriptor to be practically useful. A non-invariant such as the list of fractional coordinates of all motif points 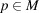 cannot distinguish between any periodic structures even under translation because all points of a motif *M* can be slightly moved along the same vector within a fixed unit cell without changing the underlying periodic structure in the sense of Definition 6[Statement definition6].

The related concept of an *equivariant* means a function *E*(*S*) such that any rigid motion *f* affects *E*(*S*) in a way controlled by *f* so that 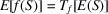, where *T_f_* depends only on *f* but not on *S*. The *invariance* means that *T_f_* is the identity.

For example, the center of mass of a finite molecule *M* is equivariant (rigidly moves together with *M*). But the center of mass of a motif *M* is not equivariant for a periodic point set *S* because a translation can push one point 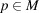 through a side face of a unit cell *U*, so the new periodic translate of *p* in the cell *U* non-equivariantly changes *M* and its center of mass.

Any linear combination of given point coordinates is equivariant under linear transformations, while invariants are much more restrictive and hence valuable. Equivariants are often used for representing inter-atomic forces by vectors that should be rigidly moved with the whole structure. Any collection of forces (one vector at every atom) can be interpreted as an ordered pair (initial structures, final structure moved by these forces).

Hence, complete invariants suffice to describe not only static structures but also any dynamics in the space of structures. Mathematical crystallography has developed many approaches to unambiguously identify a periodic structure under rigid motion, for example using a theoretically unique reduced cell (Niggli, 1928 ▸). Then any periodic structure can have standard settings in the reduced cell (Parthé *et al.*, 2013 ▸). In theory, this conventional representation is complete under rigid motion.

Fig. 1 ▸ (right) shows that almost any noise can arbitrarily scale up any reduced cell. Theorem 15 in Widdowson *et al.* (2022 ▸) states that this discontinuity under tiny perturbations holds even for lattices, which have motifs consisting of only one point.

**Figure 6:**
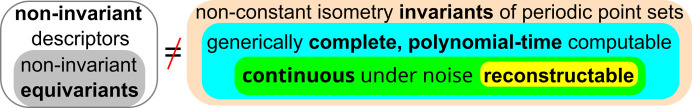
Non-invariants versus progressively harder requirements for isometry invariants, which will be all formalized in Section 5. For periodic crystals, invariants should be computable in the polynomial time for the size of the motif in order to be useful in practice.

The discontinuity of cell-based representations allows anyone to disguise a near-duplicate as a new material by making any extended cell primitive due to a slight displacement of atoms and by replacing some atoms with similar ones. To stop potential duplicates, we need continuous invariants that can quantify any (near-)duplicates in terms of a distance metric. The more practically important requirements of continuity and reconstructability in Fig. 6 ▸ will be formalized in Section 5.

## 5. Similarities versus distance metrics and continuity

Section 4 justified the importance of invariants for distinguishing periodic structures. This section formalizes the concept of continuity with respect to a distance metric. We start from the simplest non-trivial case of 2D lattices.

De Lagrange (1773 ▸) classified all lattices 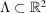 under isometry by using the quadratic form 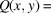

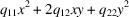
, whose coefficients are expressed via a basis *v*_1_, *v*_2_ of a lattice Λ by the formulae 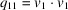, 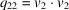 and 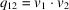. The extra conditions 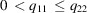 and 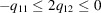 guarantee the uniqueness of the form *Q*. The corresponding basis of Λ is called *reduced* and is unique under the isometry of 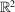 but not under rigid motion because the bases *v*_1_ = (1, 0), 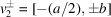 for 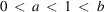 have the same reduced form 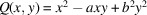 and generate lattices that are mirror images and not related by the rigid motion of 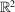.

In a more geometric approach, Selling (1874 ▸) and later Delone *et al.* (1934 ▸) added to any basis **v**_1_, **v**_2_ of 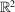, the extra vector 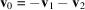 and the restriction that all pairwise angles between these vectors are *non-acute*, which means 90° or more. More recently, Conway & Sloane (1992 ▸) called such a collection 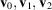 an *obtuse superbase*. This name is justified by the fact that any vector 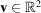 can be written as 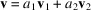 for unique 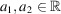 in a basis **v**_1_, **v**_2_ and also as 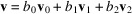 for unique 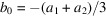, 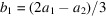 and 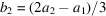 so that 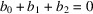.

**Figure 7:**
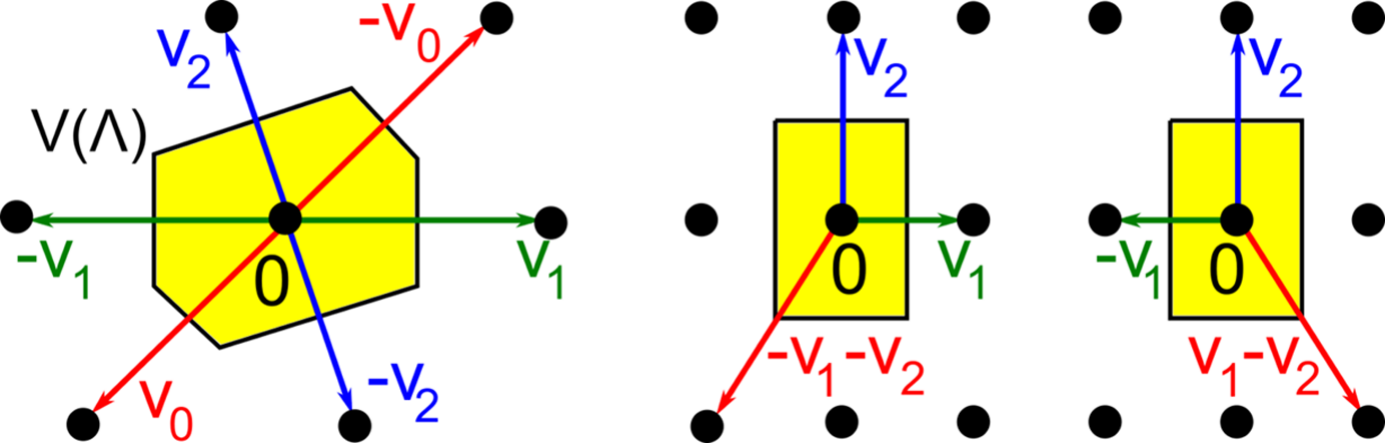
Any lattice 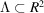 has an obtuse superbase of basis vectors 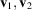 with 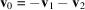 and 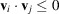 for distinct 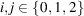, which is unique under isometry, but not under rigid motion (for the rectangular lattice on the right).

While any lattice in 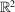 has infinite non-isometric bases [see Fig. 1 ▸ (left)], its obtuse superbase is unique up to isometry. Indeed, any non-rectangular lattice 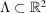 has only two opposite superbases 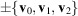, which are related by the twofold rotation around 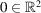, and whose all six vectors are orthogonal to the boundary of the hexagonal Voronoi domain 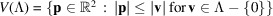 in Fig. 7 ▸ (left) (see Voronoi, 1908 ▸). All obtuse superbases of a rectangular lattice are related by reflections and are not unique under rigid motion. Fig. 7 ▸ (right) shows two obtuse superbases (mirror images) for **v**_1_ = (*a*, 0), **v**_2_ = (0, *b*) and **v**_0_ = (−*a*, −*b*).

Definition 8 (root invariant{\rm RI}(\Lambda){\rm \ of \ a \ lattice} \Lambda\subset{\bb R}^{2}) Let a lattice 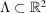 have an obtuse superbase **v**_0_, **v**_1_, **v**_2_, so that **v**_1_, **v**_2_ generate Λ, 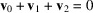 and 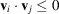 for all distinct 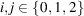. Write the *root products*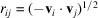 in increasing order 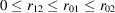, which might re-order the vectors 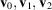 without changing Λ. The *root invariant* is the ordered triple 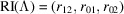, where only *r*_12_ can be 0.

Theorem 4.2 in Kurlin (2022*b* ▸) proved that 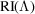 is a complete invariant of all lattices 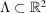 under isometry, also under rigid motion after enriching 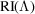 with a sign of orientation. The key advantage of 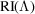 in comparison with a reduced basis is the continuity under perturbations. In Kurlin (2022*b* ▸), figure 4 explains the discontinuity of reduced bases, while theorems 7.5 and 7.7 prove the bi-continuity of the root invariant 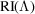.

**Figure 8:**
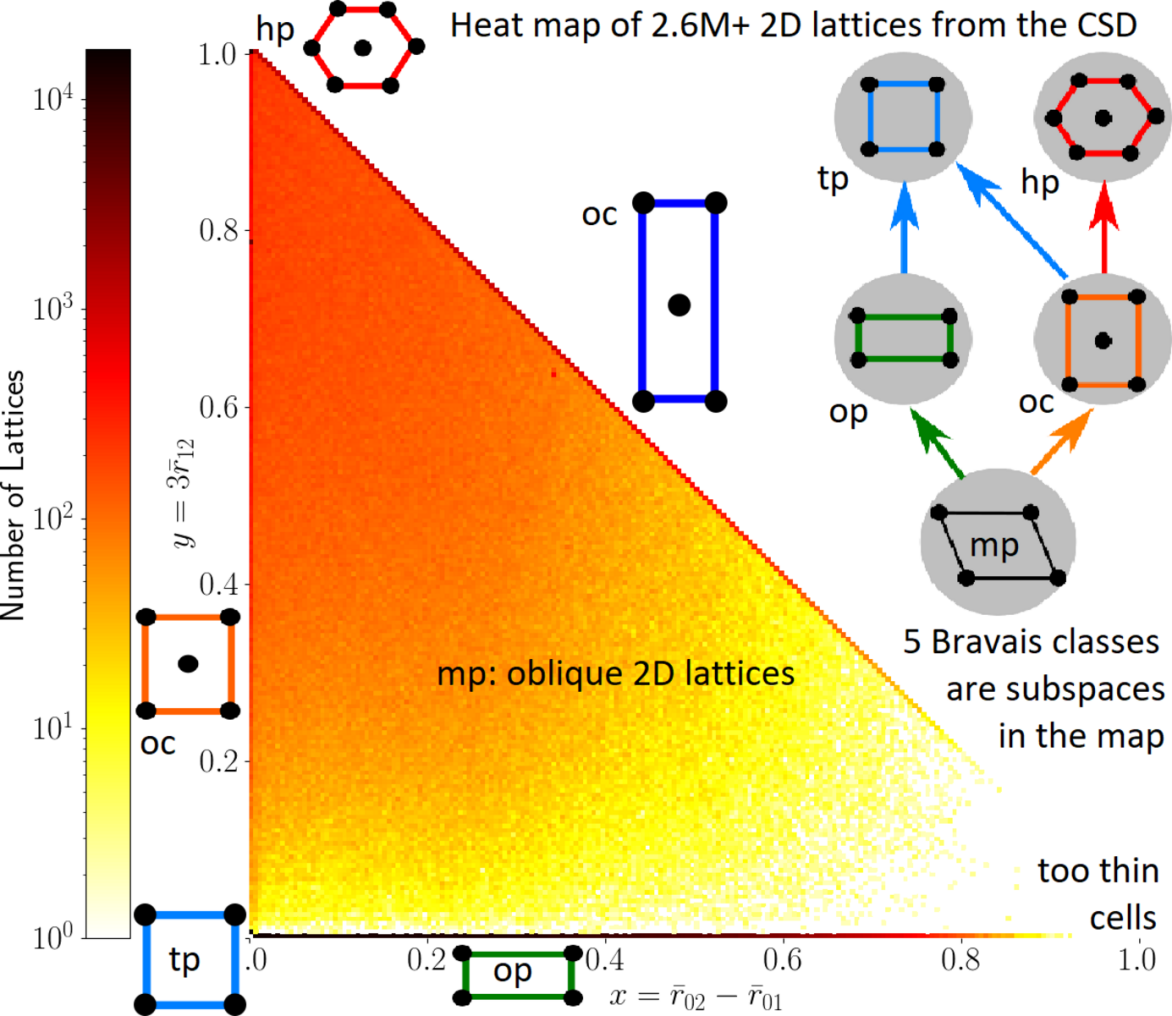
For each crystal in the CSD with a given basis 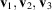, we took three lattices generated by the bases 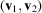, 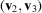 and 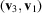. The resulting 2.6 million+ 2D lattices populate a triangle continuously expanding the tree of Bravais classes. The color indicates a logarithmically scaled number of lattices whose invariants are close to (*x*, *y*), see the earlier version in figure 9 of Bright *et al.* (2023*b* ▸).

Fig. 8 ▸ visualizes the continuous space of all 2D lattices under isometry composed (for simplicity) with uniform scaling, which maps each root product to 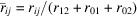. Since 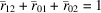, we can use only two independent coordinates 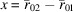 and 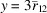 which define the *quotient triangle*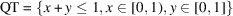. Any rectangular lattice 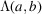 with an obtuse superbase **v**_1_ = (*a*, 0), **v**_2_ = (0, *b*), **v**_0_ = (−*a*, −*b*) for 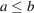 has 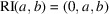 and 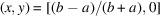. All square lattices with *a* = *b* are represented by the origin (*x*, *y*) = (0, 0). The point (1, 0) is excluded as a limit case of lattices with infinitely thin and long cells.

In summary, all classes of 2D lattices under isometry and uniform scaling are in a 1–1 bi-continuous correspondence with all points in the quotient triangle QT. The Bravais classes of square and hexagonal lattices are the points (0, 0) and (0, 1), respectively. The Bravais class of centered rectangular lattices consists of two boundary edges (without endpoints): the hypotenuse *x* + *y* = 1 and vertical side *x* = 0, 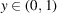.

Any continuous path in QT is realized as a continuous deformation of lattices. For example, the unit square lattice Λ_0_ with the obtuse superbase (3, 0), (0, 3), (−3, −3) and 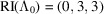 can be continuously deformed into the hexagonal lattice Λ_1_ with the obtuse superbase 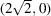, 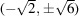 and 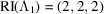 along the vertical side *x* = 0, 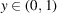 through the lattices Λ_*y*_ with 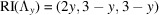 and the bases 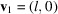 and 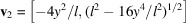, where 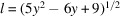 and *y* continuously moves from 0 to 1.

Fig. 8 ▸ contrasts the discrete tree of five Bravais classes of 2D lattices with the continuous map on the quotient triangle QT. Although every orthorhombic crystal from the CSD is represented by three rectangular lattices (on three pairs of reduced basis vectors), about 45% of all resulting lattices are oblique and continuously fill the interior of QT apart from the sparse corner close to (1, 0), where lattices have very thin and long primitive unit cells. All non-generic lattices occupy lower-dimensional subspaces in the continuous space of lattices.

One can define many continuous distances between points in the quotient triangle QT in Fig. 8 ▸, hence between classes of 2D lattices under isometry and uniform scaling. Section 5 in Kurlin (2022*b* ▸) gave closed-form expressions for metrics between root invariants and section 6 quantified deviations from symmetry by continuous chiral distances (see Bright *et al.*, 2023*a* ▸).

Any lattice in 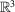 has an obtuse superbase, which is unique under isometry only for generic lattices whose Voronoi domain is a truncated octahedron. Lemmas 4.1–4.5 in Kurlin (2022*a* ▸) explicitly described all non-isometric obtuse superbases for the five Voronoi types of 3D lattices. These results led to a complete root invariant of lattices under isometry in 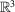 in Kurlin (2022*a* ▸). The root invariant of a 3D lattice requires complicated continuous distances satisfying the metric axioms in Definition 9[Statement definition9] below and will appear in a forthcoming work.

The even more general case of periodic point sets needs a metric satisfying the axioms below. This metric is a distance between two objects, not a numerical property of a single object.

Definition 9 (distance metric) For any objects under an equivalence relation 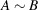 from Definition 1[Statement definition1], a distance metric 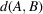 is a function satisfying these axioms:

(1) *coincidence*: 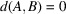 if and only if 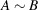;

(2) *symmetry*: 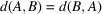 for any objects *A*, *B*;

(3) 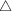
*inequality*: 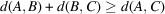 for any *A*, *B*, *C*.

The positivity property 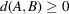 follows the axioms above. A metric is needed to formalize the continuity of invariants in Problem 10[Statement enun10] below. Though classical crystallography theoretically achieved the completeness of cell-based invariants, Problem 10[Statement enun10] asks for more practically important invariants that have no discontinuities at boundaries of 230 (or any other number of) classes in the fully connected crystal universe.

Problem 10 (isometry classification of periodic structures) Find a function *I* on all periodic point sets 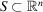 satisfying the following practically important conditions:

(a) *invariance*: if 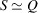 are isometric, then 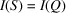;

(b) *completeness*: if 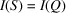, then 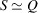 are isometric;

(c) *continuity*: there is a metric *d* satisfying the axioms of Definition 9[Statement definition9] under isometry and the 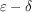 continuity below: for any 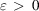 and a periodic point set *S*, there exist *C* and 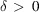 such that if *Q* is obtained by perturbing any point of *S* up to δ in Euclidean distance, then 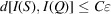;

(d) *reconstructability*: any periodic point set 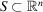 can be reconstructed (uniquely up to isometry) from its invariant *I*(*S*);

(e) *computability*: the invariant *I*, metric *d* and reconstruction of 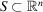 can be obtained in polynomial time of the motif size from a suitably reduced basis of *S* and motif points in this basis.

Due to the coincidence axiom of a metric in Definition 9[Statement definition9], the equality *I*(*S*) = *I*(*Q*) in the completeness condition [Statement enun10](b) of Problem 10 is best checked as 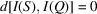. If computability condition [Statement enun10](e) of Problem 10 is missed, one impractical invariant *I*(*S*) satisfying all other conditions can be defined as the isometry class of all (infinitely many) periodic point sets isometric to *S*. We assume that a periodic point set *S* is given with a reduced basis such as the Niggli basis in 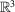 or the Minkowski basis in a higher dimension *n* since lattice reductions can be slow for 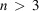 (see Nguyen & Stehlé, 2009 ▸).

The 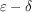 continuity condition [Statement enun10](c) of Problem 10 is a classical but weak version of continuity. The stronger *Lipschitz* continuity states that *C* and δ are independent of *S* and ɛ, so if *Q* is ɛ-close to *S*, then 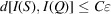, where a constant δ was absorbed by 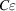.

For 2D lattices Λ, theorem 7.5 in Kurlin (2022*b* ▸) proved the intermediate Hölder continuity, stating that if the coordinates of the basis vectors of Λ are perturbed up to ɛ, the root invariant 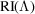 changes up to 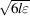 in the Euclidean metric, where *l* is the maximum length of given basis vectors of Λ.

The stronger Lipschitz continuity (without the factor 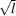) seems unrealistic for lattices because the rectangular lattices with the ɛ-close bases 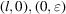 and 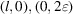 can substantially differ even by unit-cell areas 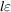 and 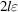 whose difference 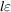 can be arbitrarily large if *l* has no upper bound.

**Figure 9:**
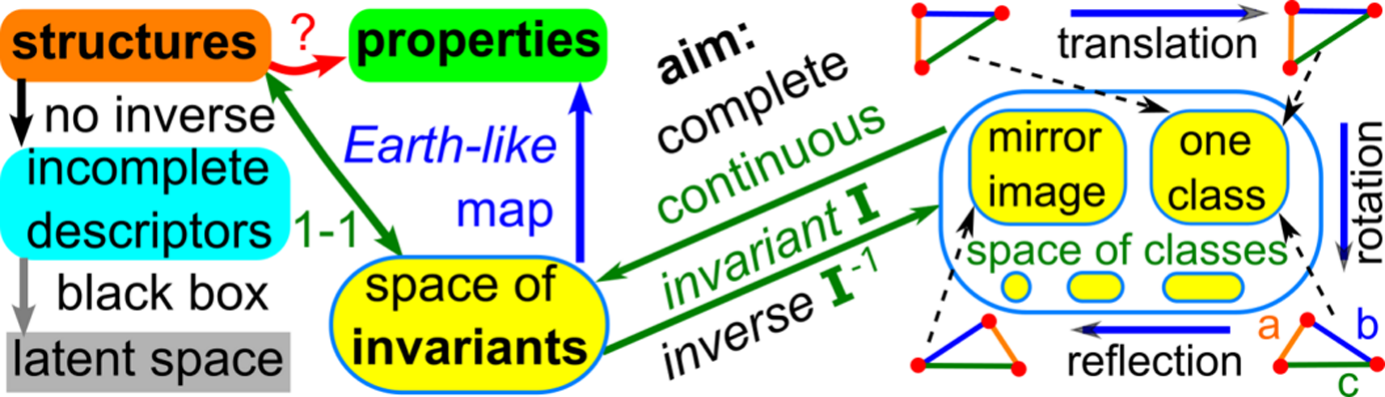
To explain the structure–property relations, a crystal structure *S* with infinitely many representations under isometry should be bijectively mapped by a complete and continuous invariant *I* to the space of invariants so that any image *I*(*S*) can be efficiently inverted back to a representative crystal 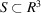.

Fig. 9 ▸ visualizes the advantages of invariants that satisfy all the conditions of Problem 10[Statement enun10]. In the past, incomplete, discontinuous or non-invariant descriptors mapped periodic crystals to latent spaces (image spaces of descriptor functions).

The *non-invariance* (existence of false negatives) means that the same crystal structure maps to different points, which makes the problem of distinguishing structures even harder. The *incompleteness* (existence of false positives) means that non-isometric structures map to the same point, which leaves no chance to reconstruct a correct crystal. The *discontinuity* under tiny atomic displacements means that near-duplicates can appear very distant in the latent space.

All the conditions of Problem 10[Statement enun10] guarantee that a required invariant *I* is a bijective and continuous map from the space of crystal structures to the space of invariant values. The inverse map *I*^−1^ reconstructs any periodic point set *S* from *I*(*S*).

## 6. Conclusions: the practical importance of definitions

This section summarizes the progress in developing invariants that satisfy the conditions of Problem 10[Statement enun10]. The root invariant from Definition 8[Statement definition8] satisfies all conditions of Problem 10[Statement enun10] for all 2D lattices even with the stronger Hölder continuity (instead of the weaker 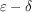 continuity) under rigid motion, which is stronger than isometry. For 3D lattices, Kurlin (2022*a* ▸) defined a complete isometry invariant whose continuity under perturbations is being finalized.

Past approaches defined metrics between lattices that allowed only slow or approximate computations. Some of these theoretical metrics were proved to be continuous for isometry classes of lattices in any dimension (Mosca & Kurlin, 2020 ▸).

In Widdowson & Kurlin (2022 ▸), for arbitrary periodic point sets *S* in 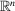, definition 3.3 defined the *Pointwise Distance Distribution*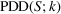, where *k* is the number of neighbors taken into account for any point in a motif. Theorem 4.3 proved the Lipschitz continuity, stating that perturbing any atom up to ɛ changes 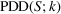 only up to 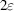 in a suitable metric. Theorem 4.4 proved that 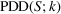 is *generically complete* in the sense that almost any periodic structure 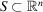 (outside singular subspaces of measure 0) can be reconstructed from a lattice of *S* and 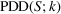 with an explicit upper bound on *k* depending on a given unit cell and motif of *S*. Hence PDD can be considered a DNA-style code that uniquely identifies almost any real periodic crystal. PDD is stronger for periodic crystals than DNA, which allows identical twins (about 0.3% among humans) with indistinguishable DNA (see Osterman *et al.*, 2022 ▸).

In practice, 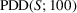 distinguished all (more than 660 000) different periodic crystals in the Cambridge Structural Database (CSD) through more than 200 billion pairwise comparisons, which were completed within two days on a modest desktop. Section 6 in Widdowson & Kurlin (2022 ▸) lists several pairs that turned out to be near-duplicate CIFs, where all numbers (unit-cell parameters and fractional coordinates) were identical almost to the last decimal place, but one atom was replaced with a different one, *e.g.* Cd with Mn in the pair JEPLIA versus HIFCAB. The integrity office of the Cambridge Crystallographic Data Centre and all other crystallographers who looked at these previously unknown near-duplicates agreed that such an atomic replacement should more substantially perturb the geometry of atomic centers, so five journals are investigating the data integrity of the underlying publications.

A forthcoming paper will extend PDD invariants to distinguish all known pairs of *homometric* crystals that (by definition) have the same (infinite) list of all interatomic distances. We conjecture that the extended invariants are theoretically complete for all periodic point sets under isometry in any Euclidean 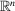.

The comparisons above use only geometry of atomic centers without chemical elements. After excluding the unrealistic duplicates found in the CSD, the PDD invariants mapped all non-isometric crystal structures to non-isometric periodic structures, where each atom is replaced with a zero-sized point.

**Figure 10:**
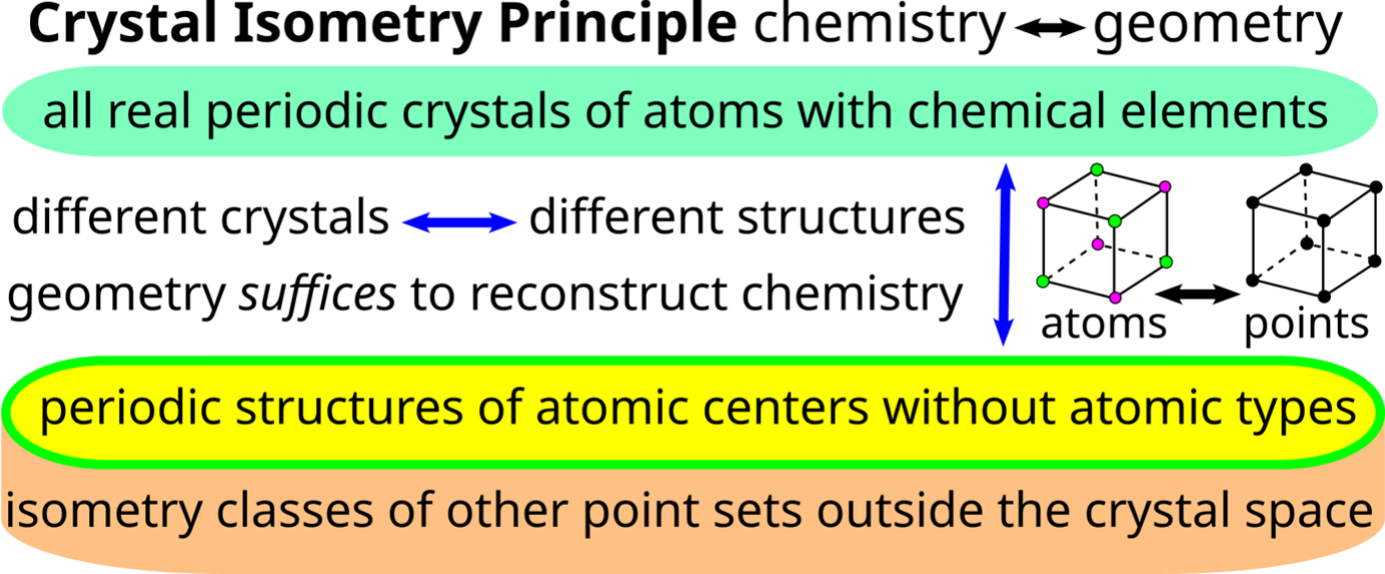
The *crystal isometry principle* states that all atomic types in real periodic crystals can be reconstructed from the geometry of atomic centers given with enough precision, first stated in section 6 of Widdowson *et al.* (2022 ▸) and inspired by Feynman’s visual hint in Fig. 4 ▸, see Figure 1-7 in Feynman *et al.* (1971 ▸).

Since this map is injective, the more important conclusion is the *crystal isometry principle* (CRISP) which states that any real periodic structure has a unique location in a common *Crystal Isometry Space* of all periodic structures (isometry classes of periodic point sets) independent of symmetry, see Fig. 10 ▸.

Hence, in principle, all atomic types in a real periodic crystal can be reconstructed from a sufficiently precise geometry of their atomic centers. The Eureka moment for this insight happened in May 2021 when the second author was reading Richard Feynman’s first lecture ‘Atoms and motion’ (see Fig. 4 ▸) with the table of seven cubic crystals whose chemistry can be reconstructed from the only geometric parameter *d* (smallest inter-atomic distance) known to two decimal places.

The crystal isometry principle does not claim that any periodic point set gives rise to a real periodic crystal because inter-atomic distances cannot be arbitrary. However, any newly discovered periodic crystal will appear in the same continuous universe, where all known crystals are already visible. Fig. 8 ▸ showed a map of 2D lattices under isometry and uniform scaling. Continuous maps of the CSD and other databases in invariant coordinates were presented at the IUCr congress (see Kurlin, 2023 ▸) and will be discussed in future work.

While the realizability of root invariants by lattices in two and three dimensions has been established in Kurlin (2022*b* ▸,*a* ▸), we keep working on the harder problem of realizability of PDD invariants. The implemented application of PDD is the ultra-fast detection of (near-)duplicates in structural databases. The final sections in Widdowson *et al.* (2022 ▸) and Widdowson & Kurlin (2022 ▸) reported over a dozen such pairs in the CSD. Another forthcoming work will report less obvious (near-)duplicates in the CSD and many more duplicates in the Crystallography Open Database (COD), Inorganic Crystal Structure Database (ICSD), Materials Project and others.

The most important practical impact of CRISP is the scientific barrier for ‘paper mills’ and ‘duplicate generators’ that can output thousands and even millions of ‘predicted’ and sometimes ‘synthesized’ materials by disguising known structures as new by tiny perturbations of cell parameters and atomic coordinates (structure factors or other experimental data if needed) to scale up a primitive cell, and finally by changing some non-standard chemical elements to their suitable neighbors in the periodic table. Google’s example below shows that even big numbers cannot mask (near-)duplicates that we can filter out by numbers in given CIFs even before computing invariants.

The paper finishes by describing embarrassing coincidences in Google’s GNoME database of 384 398 ‘stable’ structures in Google (2023 ▸) predicted by expensive DFT optimization (Mardirossian & Head-Gordon, 2017 ▸). The following *crystal bug test* can substantially reduce further invariant computations for such a large database. Ordering all CIFs by the unit-cell volume detected many thousands of pairs of CIFs in GNoME that have identical volumes to all (eight) decimal places (digits).

Other colleagues found some duplicates after ordering all CIFs by file sizes in bytes, but filtering by the unit-cell volume is more justified. Further filtering by six parameters (three lengths and three angles) of a unit cell found 30 000+ CIFs with identical unit cells, again with all given digits.

Table 1 ▸ summarizes more hard-to-explain coincidences. The CIFs with GNoME IDs 4135ff7bc7, 6370e8cf86, c6afea2d8e and e1ea534c2c are identical texts (symbol-by-symbol). The supporting information contains an Excel table listing more than a thousand pairs of identical CIFs. If chemical elements are ignored, GNoME has 1481 pairs of CIFs with all equal numbers (unit-cell parameters and fractional coordinates). If we round all numbers to four and two decimal places for the precision of 10^−4^ Å and 10^−2^ Å, respectively, the last two columns in Table 1 show many more groups of CIFs that become numerically identical to each other. Table 2 ▸ shows chemical compositions for the three largest groups of CIFs.

The first part of Table 2 ▸ shows that GNoME contains a group of nine CIFs, where all numbers are equal (with all decimal places) but chemical compositions differ by one or two atoms. For example, Dy, Y, Ho and Tb are often swapped. If all numbers are rounded to two digits, one more CIF (a18d30a9fc) joins the group of duplicates, where Ru is replaced with Re. So comparisons by unit-cell parameters and fractional coordinates can help to filter out obvious (near-)duplicates even in big data.

This paper clarifies the concept of a periodic crystal in terms of an ordered basis whose re-ordering creates ambiguity or discontinuity in Fig. 2 ▸. Definitions 2[Statement definition2], 3[Statement definition3] and 4[Statement definition4] are visually summarized in Fig. 3 ▸. Rigid motion (or slightly weaker isometry) is motivated as the strongest equivalence between crystals whose structures are determined in a rigid form. The practical importance of distinguishing near-duplicates in major structural databases requires us to define a periodic (crystal) structure as an *equivalence class under rigid motion*. Any deviations from an ideal rigid matching should be continuously quantified in terms of a distance metric satisfying all axioms and at least the classical 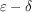 form of continuity.

As a visual summary, Fig. 6 ▸ highlights the importance of invariants versus non-invariant descriptors. Fig. 5 ▸ explains that similarities based on single (hence non-invariant) finite subsets are hard to justify for periodic structures. In the past, crystallography developed conventional representations based on reduced that can be considered complete isometry invariants in theory.

Now computational resources are used for generating millions of structures, many of which turn out to be near-duplicates. Hence Problem 10[Statement enun10] has become the important scientific barrier for paper ‘milling’ by validating any newly discovered crystals versus all known ones. Future work will extend PDD to a full solution of Problem 10[Statement enun10]. The crystal isometry principle and underlying invariants were used for property predictions in the literature (Ropers *et al.*, 2022 ▸; Balasingham *et al.* 2024*a* ▸,*b* ▸) and were presented at the IUCr congresses in 2021 and 2023, the European Crystallographic Meeting 2022, the BCA annual meetings 2022–2024, and MACSMIN 2021–2023 (Mathematics and Computer Science for Materials Innovation).

## Supplementary Material

Zipped tables listing more than a thousand pairs of identical CIFs from GNoME. DOI: 10.1107/S2052252524004056/gq5017sup1.zip

## Figures and Tables

**Table 1:** Coincidences across all CIFs in the GNoME database of 384 398 publicly available CIFs (Google, 2023 ▸) The first column shows the sizes of the found groups whose CIFs are (near-)duplicates. Columns 2–5 count fully identical (symbol-by-symbol) CIFs, the CIFs where all numbers (unit-cell parameters and fractional coordinates) coincide with all digits (at least six), then CIFs where all numbers coincide up to four and two digits, respectively. The last row counts the total number of the involved CIFs. The largest groups are listed in Table 2 ▸

Group size: No. of CIFs	CIFs are identical texts	All numbers coincide	Rounding to four digits	Rounding to two digits
10	0	0	0	1
9	0	1	1	0
7	0	1	1	2
6	0	2	2	4
5	0	2	3	18
4	1	8	12	92
3	43	72	96	670
2	1089	1481	1932	7856
Total	2311	3248	4243	18228

**Table 2:** The largest groups of (near-)duplicates from Table 1 ▸ in the GNoME database

GNoME ID	Chemical formula	All numbers in CIFs coincide	Numbers coincide up to two digits
082738d51d	Dy_1_Y_6_Ho_13_Cd_6_Ru_2_	In a group of 9	In a group of 10
1fba8c028f	Dy_2_Y_4_Ho_14_Cd_6_Ru_2_	9	10
39fe92e2ee	Tb_2_Y_4_Ho_14_Cd_6_Ru_2_	9	10
6d47ae3d9f	Tb_3_Y_3_Ho_14_Cd_6_Ru_2_	9	10
703ed1d823	Tb_6_Ho_14_Cd_6_Ru_2_	9	10
78fcd9d814	Tb_1_Y_5_Ho_14_Cd_6_Ru_2_	9	10
976f8cb279	Y_6_Ho_14_Cd_6_Ru_2_	9	10
a30e9d8c9b	Tb_5_Y_1_Ho_14_Cd_6_Ru_2_	9	10
b8c0e953e2	Tb_4_Y_2_Ho_14_Cd_6_Ru_2_	9	10
a18d30a9fc	Tb_6_Ho_14_Cd_6_Re_2_	In a group of 1	10
06eb60e958	Li_2_Tb_2_Ho_4_Hg_8_	In a group of 7	In a group of 7
9762be0ec6	Li_2_Tb_2_Dy_4_Hg_8_	7	7
ab336b54ee	Li_2_Tb_2_Er_4_Hg_8_	7	7
aed8780f34	Na_2_Tb_2_Lu_4_Hg_8_	7	7
c2236e05de	Na_2_Tb_2_Dy_4_Hg_8_	7	7
ca1d14568f	Na_2_Tb_2_Tm_4_Hg_8_	7	7
d9eab4539b	Li_2_Tb_2_Y_4_Hg_8_	7	7
02c4cb55a6	Tb_5_Dy_15_Cd_6_Ru_2_	In a group of 6	In a group of 7
0affe9c149	Tb_2_Dy_18_Cd_6_Ru_2_	6	7
100cfdfdef	Tb_3_Dy_17_Cd_6_Ru_2_	6	7
877c190805	Tb_4_Dy_16_Cd_6_Ru_2_	6	7
9ce48821cb	Dy_20_Cd_6_Ru_2_	6	7
b9e4b78276	Tb_1_Dy_19_Cd_6_Ru_2_	6	7
cf7af6f79f	Dy_9_Y_6_Ho_5_Cd_6_Ru_2_	In a group of 1	7
